# Site‐Specific Characterization and Quantification of Facial Corticosteroid Addiction Dermatitis: A Multimodal Nomogram Integrating High‐Frequency Ultrasonography and Transepidermal Water Loss

**DOI:** 10.1111/srt.70388

**Published:** 2026-10-12

**Authors:** Jun Wang, Ji Tao Gui, Rong Yu, Hai Rui Wang, Yang Hu, Ting Li

**Affiliations:** ^1^ Department of Ultrasound, Nanxiang Branch Shanghai Jiao Tong University School of Medicine Shanghai China; ^2^ Department of Dermatology, Ruijin Hospital, Nanxiang Branch Shanghai Jiao Tong University School of Medicine Shanghai China

**Keywords:** Facial corticosteroid addiction dermatitis, generalized estimating equations, nomogram, power Doppler imaging (PDI), regional heterogeneity, subepidermal low‐echogenic band (SLEB), topical corticosteroid withdrawal, transepidermal water loss (TEWL)

## Abstract

**Background:**

The evaluation of facial corticosteroid addiction dermatitis (FCAD) lacks objective criteria, and existing clinical evaluation methods largely ignore regional facial heterogeneity. This study aimed to develop a site‐adjusted multimodal nomogram integrating 22‐MHz high‐frequency ultrasonography (HFUS) and transepidermal water loss (TEWL) to serve as an adjunctive quantitative assessment tool to quantify the FCAD pathological state objectively.

**Materials and Methods:**

In this prospective case‐control study, 279 facial sites (119 lesional, 160 healthy) from 40 patients with FCAD and 40 healthy controls (HCs) were evaluated. Generalized estimating equations (GEE) were employed to adjust for the intra‐subject clustering effect of multisite data.

**Results:**

Multivariable GEE identified four independent predictors for FCAD: marked blood flow on power Doppler imaging (PDI) (adjusted odds ratio [aOR] = 19.46, *p* < 0.001), subepidermal low‐echogenic band (SLEB) thickness (aOR = 1.76 per 0.01 mm increase, *p* = 0.005), TEWL (aOR = 1.11, *p* = 0.002), and the chin site (vs. forehead, aOR = 0.35, *p* = 0.009). The resulting nomogram achieved an area under the curve (AUC) of 0.905, significantly outperforming a single‐parameter vascular model (AUC = 0.821, *p <* 0.05). At the optimal probability cutoff, sensitivity was 85.7% and specificity was 86.9%.

**Conclusion:**

Integrating vascular, structural, and functional dimensions with anatomical site adjustments establishes a robust baseline for characterizing FCAD. This multimodal nomogram is intended for use as an adjunctive tool for quantitative assessment and disease monitoring, facilitating a critical shift from subjective clinical evaluation to precise, quantitative imaging.

## 1 Introduction

Facial corticosteroid addiction dermatitis (FCAD) is a recalcitrant inflammatory dermatosis caused by the prolonged topical application of glucocorticoids. This chronic exposure induces cutaneous dependence, leading to a rebound or exacerbation of primary skin lesions upon discontinuation, thereby compelling patients to repeatedly resume application [1]. Given its distinct clinical course and phenotype, this condition is frequently categorized in the international literature as “topical corticosteroid withdrawal” (TCW) [2], or based on morphological features, as “steroid‐induced rosacea‐like dermatitis” [3]. To maintain terminological consistency, the acronym “FCAD” is used uniformly throughout this study. Recently, driven by the misuse of corticosteroids in cosmetic products, the incidence of FCAD has increased significantly, emerging as a critical dermatological challenge globally, particularly in Asia [1].

Clinically, the hallmark of FCAD is the “rebound phenomenon.” Upon the cessation of topical steroids, patients rapidly develop diffuse erythema, papules, pustules, and telangiectasia, often accompanied by intolerable burning and pruritus [3, 4]. Despite the mounting clinical burden of FCAD, its current evaluation predominantly relies on guideline‐based clinical diagnosis, retrospective medication histories, and subjective visual dermatologic examinations. While adjunctive tools such as dermoscopy have been explored to visualize superficial vascular changes [5], this reliance on subjective assessment, coupled with a lack of objective, standardized quantitative criteria and phenotypic overlaps with conditions such as rosacea, seborrheic dermatitis, and contact dermatitis, results in substantial interobserver variability [5, 6]. While differential diagnosis remains a clinical hurdle, resolving this challenge necessitates first establishing a specific imaging baseline for FCAD that clearly distinguishes its pathological state from physiological homeostasis. Therefore, this study aims to develop a multimodal nomogram incorporating the anatomical site as a crucial predictive factor. By integrating high‐frequency ultrasonography (HFUS) and transepidermal water loss (TEWL) assessments, we seek to construct a site‐adjusted adjunctive assessment tool capable of objectively quantifying and characterizing the pathological state of FCAD across different facial regions.

Driven by advancements in dermatological imaging technologies, noninvasive bioengineering tests have become essential tools for evaluating cutaneous pathological states. Notably, 20–50 MHz HFUS provides high‐resolution visualization of cutaneous microstructures. A characteristic HFUS finding, the subepidermal low‐echogenic band (SLEB), has been validated as a sensitive imaging biomarker for dermal edema and inflammatory infiltration, correlating strongly with the severity of inflammatory dermatoses [7, 8]. Furthermore, Power Doppler Imaging (PDI) serves as a vital hemodynamic tool for detecting microvascular hyperperfusion. Concurrently, TEWL—the “gold standard” for evaluating epidermal barrier function—is widely utilized to monitor stratum corneum integrity [9]. However, prior studies on FCAD have typically evaluated these modalities in isolation [5, 24, 30], lacking a comprehensive assessment that bridges morphological structure and vascular hemodynamics with physiological function. Consequently, they fail to fully capture the complex pathophysiological alterations inherent to the disease.

Crucially, existing diagnostic models generally overlook the regional heterogeneity of facial skin, representing a significant gap in the current literature. Anatomical studies demonstrate that distinct facial subunits (e.g., the forehead, cheeks, and chin) exhibit significant physiological variations in skin thickness, sebum secretion, and barrier function [10]. Even within healthy populations, ultrasound measurements such as skin thickness and echodensity differ significantly across varying facial regions [11]. In statistical modeling, neglecting the clustering effect of multisite data derived from the same subject and resorting to simple pooling analyses inevitably leads to Type I error inflation and statistical bias. Therefore, implementing statistical strategies capable of handling intra‐subject correlations, such as generalized estimating equations (GEE), alongside site‐specific adjustments is paramount for enhancing assessment robustness. In light of this, the present study aims to integrate HFUS parameters, physiological metrics, and anatomical site characteristics to construct a multimodal nomogram. The established baseline imaging characteristics of FCAD versus healthy skin provide a robust parametric foundation for future differential diagnostic studies involving other inflammatory dermatoses. This approach will establish a quantitative imaging baseline for the pathological deviation of FCAD relative to healthy skin. Ultimately, rather than immediately pursuing complex clinical differential diagnoses, this research focuses on identifying the fundamental distinctions between the pathological and physiological states, thereby laying a robust foundation for future generalized differential models that encompass confounding conditions such as rosacea.

## 2 Materials and Methods

### 2.1 Study Design and Participants

This prospective, observational case‐control study was conducted between January 2023 and July 2025. The research protocol was approved by the Institutional Review Board of Ruijin Hospital, Nanxiang Branch (Approval No.: 2020006) and adhered to the principles of the Declaration of Helsinki [12]. All participants provided voluntary written informed consent prior to the commencement of the study. A total of 80 subjects were enrolled, comprising 40 patients with FCAD and 40 healthy controls (HCs). The FCAD cohort was predominantly female (90.0%), aligning with the established epidemiological profile of the disease. While the two groups were comparable regarding sex distribution and body mass index (BMI), a baseline difference in age was noted. This demographic disparity was subsequently addressed through rigorous covariate adjustments and propensity score matching (PSM) in the analytical phase.

#### 2.1.1 Establishment of Diagnostic Criteria

Recognizing that the current Guidelines for the Diagnosis and Treatment of Hormone‐Dependent Dermatitis (2009 Edition) [13] lack objective quantitative parameters, and noting the absence of a unified international diagnostic consensus on TCW [3, 6], we implemented a rigorous diagnostic strategy combining clinical features with an independent, blinded expert review. This framework was designed to minimize subjective bias and ensure cohort homogeneity. All suspected cases underwent initial screening based on the aforementioned guidelines, followed by independent diagnostic verification by two senior dermatologists (each with more than five years of clinical experience). Diagnostic discrepancies were resolved through adjudication by a third independent expert.

#### 2.1.2 Inclusion and Exclusion Criteria

The inclusion criteria were as follows: (1) meeting the clinical diagnosis of FCAD, defined by the continuous facial application of topical glucocorticoids or corticosteroid‐adulterated skincare products for >1 month; (2) exhibiting the “rebound phenomenon,” characterized by the recurrence or emergence of new inflammatory lesions (e.g., erythema, papules) following the cessation of the implicated products; (3) complete cessation of the suspected pathogenic products at the time of examination, accompanied by overt rebound inflammatory symptoms; and (4) aged 18 to 70 years.

The exclusion criteria were: (1) a history of primary facial dermatoses (e.g., acne vulgaris, rosacea, or seborrheic dermatitis) to eliminate nonsteroid‐induced pathological confounders; (2) pregnancy or lactation; (3) the presence of severe systemic diseases; and (4) a history of facial laser/phototherapy or excessive sun exposure within the preceding month, to mitigate potential confounding effects on cutaneous hemodynamic and barrier function assessments.

#### 2.1.3 Delineation of Data Collection

To ensure the model's specificity in identifying active pathological features, data analysis was strictly confined to clinically overt lesional areas in patients with FCAD. Consequently, macroscopically normal‐appearing, non‐lesional facial areas of the same patients were meticulously excluded from the analysis.

### 2.2 Environmental Standardization and Measurement Protocol

Given the specific environmental requirements of the distinct modalities, a two‐step sequential measurement protocol was implemented. Subjects first completed the TEWL assessment in the skin barrier laboratory, immediately followed by the HFUS examination in the ultrasound suite. Although physically separated, both rooms were regulated by the same central climate control system to maintain a constant ambient temperature (range: 19.0°C–26.0°C; mean: 25.0°C) [14]. An independent‐samples *t*‐test confirmed no significant difference in testing temperatures between the study groups (*p >* 0.05), ensuring the comparability of baseline assessment conditions.

To eliminate confounding effects from environmental and physiological fluctuations, a strict acclimatization protocol was enforced [15]:

#### 2.2.1 Cleansing and Resting

Following mild facial cleansing with water, subjects rested in a seated position within the controlled testing environment for a minimum of 20 min.

#### 2.2.2 Behavioral Control

During the acclimatization period, the face remained fully exposed. Subjects were strictly prohibited from touching their faces, engaging in vigorous physical activity, consuming hot beverages, and being exposed to direct airflow or sunlight.

#### 2.2.3 Time Bias Management

All measurements were scheduled randomly within standard outpatient clinic hours (08:00–17:00) to minimize potential systematic biases arising from circadian rhythms during intergroup comparisons.

### 2.3 Assessment of TEWL

TEWL at selected anatomical sites was quantified using a Tewameter TM 300 (Courage + Khazaka electronic GmbH, Cologne, Germany).

#### 2.3.1 Measurement Sites

Four standard anatomical loci were assessed for each subject: the glabella (Site 1), right cheek (Site 2), left cheek (Site 3), and chin (Site 4).

#### 2.3.2 Operational Protocol

The measurement probe was positioned exactly perpendicular to the skin surface, maintaining firm contact without applying excessive pressure. This precaution prevented the alteration of local microvascular circulation and the occlusion of water vapor flux. Once the reading stabilized (typically within 30–60 s), three consecutive measurements were recorded for each site, and the arithmetic mean was calculated. Results were expressed in g/m^2^h. This metric served to quantitatively evaluate the integrity of the stratum corneum permeability barrier.

### 2.4 HFUS Assessment

HFUS evaluations were performed using a MyLabTwice eHD color Doppler ultrasound scanner (Esaote S.p.A., Genoa, Italy) equipped with a 22‐MHz HFUS linear array transducer (SL3116). To eliminate interobserver variability and ensure optimal data consistency, all examinations and subsequent image parameter extractions were executed by a single senior physician with over five years of experience in dermatological HFUS. To minimize measurement variability and ensure high reproducibility, we strictly adhered to a standardized anatomical positioning protocol. Specifically, site selection was based on precise anatomical landmarks (e.g., the geometric center of the cheek, the midline of the glabella), thereby minimizing observer–dependent variations. Furthermore, to account for potential variability related to probe placement and local tissue heterogeneity, each anatomical site was evaluated by acquiring three independent ultrasound images, with the transducer lifted and repositioned between acquisitions. Crucially, to prevent mechanical compression artifacts that could artificially alter skin layer thicknesses or occlude low‐velocity microcirculation, a generous layer of ultrasound coupling gel was applied as a standoff medium. This strict “minimal‐pressure” (floating) technique ensured that the transducer did not directly compress the skin surface during any stage of image acquisition. A single morphological measurement (e.g., SLEB thickness) was subsequently extracted from each of the three distinct sonograms, and these values were averaged to calculate the final arithmetic mean. To minimize potential bias, the examining physician was strictly blinded to the subjects' detailed medical histories (e.g., duration of corticosteroid application, specific pharmacological formulations) and their corresponding TEWL measurements.

### 2.5 Morphological Parameters

**Entrance echo layer thickness**: Acknowledging the physical resolution limits of the 22‐MHz transducer, the true epidermis cannot be reliably visualized as an isolated distinct structure. Therefore, this parameter is defined on the sonogram as the distance from the epidermal entrance echo to the dermal‐epidermal junction (DEJ), more accurately corresponding to the thickness of the entrance echo layer rather than the true epidermal thickness.

**SLEB thickness**: Defined as the thickness of the hypoechoic, band‐like region emerging within the papillary dermis. This serves as an imaging correlate for inflammatory edema in the superficial dermis. In the raw dataset, this parameter specifically corresponds to the measurement of the superficial dermis (i.e., the SLEB).

**Hemodynamic parameters**: PDI was employed to evaluate cutaneous microcirculation. A low wall filter and a low pulse repetition frequency (PRF: 750 Hz) were configured to optimize the detection of low‐velocity microvascular blood flow. To quantify the degree of vascularity, we utilized the Adler grading system, specifically calibrated for facial anatomy [16]. Recognizing the extreme sensitivity of PDI to low‐velocity microvascular flow, alongside the inherent hypervascularity characteristic of facial anatomical structures, we applied a strict physiological calibration to the grading criteria. The specific standards were defined as follows:

**Grade 0 (Absent)**: No detectable blood flow signals.
**Grade 1 (Minimal)**: Minimal blood flow; only one to two isolated, punctate signals are visible.
**Grade 2 (Moderate)**: Moderate blood flow; short linear vessels or several scattered small vascular signals are visible, representing the common physiological baseline of blood flow inherent to healthy facial skin.
**Grade 3 (Marked)**: Marked blood flow signals; presenting as an interconnected reticular or branching pattern of vasodilation.

### 2.6 Statistical Analysis

Statistical analyses were performed using SPSS 27.0 (IBM Corp., Armonk, NY) and R software (Version 4.5.2). Continuous variables are expressed as the mean ± standard deviation (SD); intergroup comparisons were conducted using the independent‐samples *t*‐test or the Mann–Whitney U test, depending on the data distribution. Categorical variables are presented as frequencies (percentages), and intergroup comparisons were evaluated using the chi‐square (*χ^2^*) test or Fisher's exact test, as appropriate.

#### 2.6.1 Variable Preprocessing

Prior to model construction, key variables underwent systematic preprocessing. To assess the clinical interpretability of SLEB thickness, “pathological SLEB thickening” was defined as a measurement exceeding the upper limit of the reference range established by the healthy control group (i.e., mean + 2 SD, ≥ 0.12 mm); differences between groups were subsequently compared using the *χ^2^* test. Furthermore, PDI blood flow signals were binarized. Adler Grades 0–2 were categorized as “physiological background flow,” a classification that aligns with the inherently rich baseline vascularity of facial anatomy. Conversely, Grade 3 was delineated as “rebound vasodilation,” a hallmark indicating the pathological state of the acute FCAD withdrawal phase. This dichotomization aimed to filter out noise from physiological blood flow fluctuations, thereby substantially enhancing the model's specificity in identifying active lesions.

#### 2.6.2 Confounding Adjustment and Sensitivity Analysis

Baseline analyses revealed that the FCAD group was significantly older than the healthy control group (*p =* 0.007). Consequently, age was systematically incorporated into all subsequent multivariable models as a potential confounder to eliminate the impact of demographic disparities on the model's predictive efficacy. To further mitigate potential bias introduced by these age differences, a PSM sensitivity analysis was executed [17]. Utilizing age and BMI as covariates, a 1:1 nearest‐neighbor matching algorithm was applied with a caliper set to 0.02. Following matching, a sub‐cohort with a well‐balanced age distribution was established, and intergroup differences in ultrasonographic and physiological parameters were reevaluated to robustly verify the primary findings.

### 2.7 Model Construction and Validation

Given that data were collected from four distinct anatomical sites per subject, the dataset exhibited a significant intra‐subject clustering effect. To properly account for this correlation, GEE were employed for the statistical analysis [18]. During model construction, the working correlation matrix was evaluated based on the Quasi‐likelihood under the Independence model Criterion (QIC) and model convergence. Due to the unstable convergence exhibited by the “unstructured” and “exchangeable” matrices within the existing data, an “independent” working correlation matrix was ultimately selected. To ensure valid inferences under this assumed independent correlation structure and to fully adjust for the intra‐subject clustering effect, robust standard errors were utilized for all parameter estimations.

Rather than constructing separate models for each individual site, we incorporated the “anatomical site” as an independent covariate within a unified GEE model to adjust for baseline heterogeneity across different facial subunits. Furthermore, preliminary analyses examined interaction terms between the site and key predictors (e.g., SLEB, PDI); as these lacked statistical significance (*p >* 0.05), they were excluded in favor of a parsimonious model comprising only main effects. Based on the multivariable GEE analysis, a multimodal quantitative assessment nomogram integrating anatomical site, SLEB, PDI, TEWL, and age was constructed [19].

Model performance was evaluated across the following three dimensions:

#### 2.7.1 Discrimination

The discriminative ability of the nomogram was quantified using the area under the receiver operating characteristic (ROC) curve (AUC). The DeLong test was utilized to compare AUC differences between the multimodal model and single‐parameter models. The optimal pathological identification threshold was determined by maximizing the Youden index (sensitivity + specificity ‐ 1). At this specific threshold, the sensitivity, specificity, positive predictive value (PPV), and negative predictive value (NPV) were calculated.

#### 2.7.2 Calibration

Internal validation was performed using a cluster‐based bootstrap resampling method (*B* = 1000 iterations) via the ‘rms’ package in R. This specific methodology accounts for the clustering effect of multisite data derived from the same subject. An optimism‐corrected C‐index was calculated, and a calibration curve was plotted to evaluate the concordance between the predicted probabilities and the actual observed probabilities.

#### 2.7.3 Clinical Utility

Decision curve analysis (DCA) was conducted to evaluate the net clinical benefit of the model across a diverse range of threshold probabilities [20]. All statistical tests were two‐sided, and a *p*‐value < 0.05 was considered statistically significant.

## 3 Results

### 3.1 Study Population and Baseline Characteristics

This study enrolled a total of 80 subjects, comprising 40 patients with FCAD and 40 HCs. To construct a specific quantitative model for active dermatitis, the analytical scope was strictly restricted to clinically overt lesional areas. Consequently, 41 macroscopically normal‐appearing, non‐lesional (subclinical) sites in the FCAD group were excluded from the current analysis. The final dataset incorporated into the modeling analysis comprised 279 facial sites (119 lesional sites vs. 160 healthy sites). Notably, the anatomical distribution of the lesions exhibited a significant imbalance, consistent with the typical clinical feature of “relative perioral sparing” in FCAD: the majority of lesions were concentrated on the cheeks (*n* = 74), followed by the forehead (*n* = 25), while only 20 sites in the chin region were included in the analysis (accounting for 16.8%). This variance in sample distribution, driven by the natural topographic pattern of the disease, was statistically adjusted for within the generalized estimating equation (GEE) model. A comprehensive visual summary of the study design, participant enrollment, site‐specific exclusion, and multimodal assessment pipeline is illustrated in Figure 1.

**FIGURE 1:**
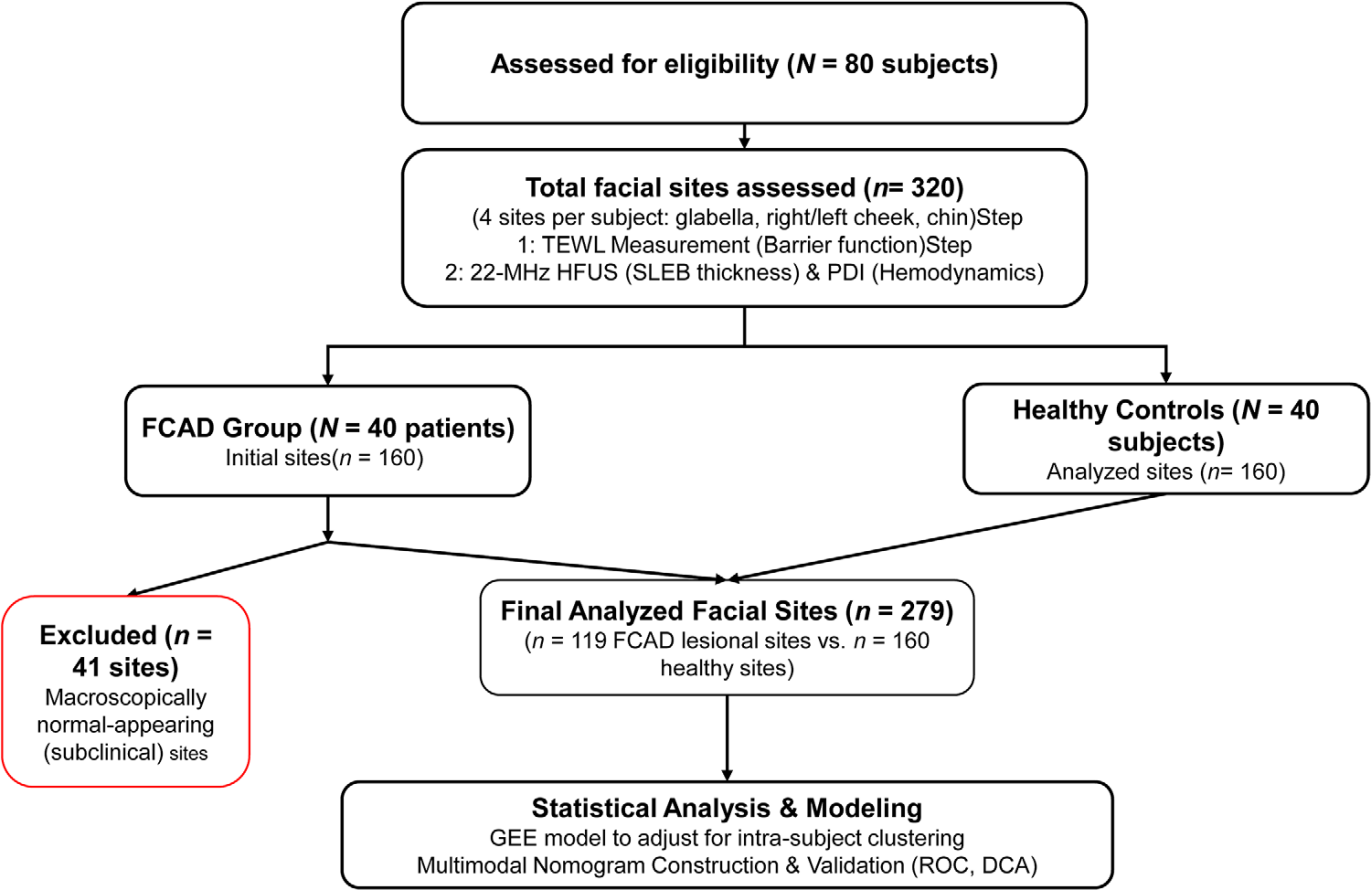
Study workflow and multimodal assessment pipeline. A total of 80 subjects were enrolled, yielding 320 facial evaluation sites. After the exclusion of 41 macroscopically normal‐appearing sites in the FCAD group, a final dataset of 279 sites was analyzed using a sequential multimodal protocol and GEE.

As presented in Table 1, there were no statistically significant differences in gender distribution or BMI between the two groups (*p >* 0.05). The mean age of the FCAD cohort was significantly higher than that of the healthy control group (39.1 ± 10.3 years vs. 33.8 ± 6.4 years; mean difference, 5.3 years; *p =* 0.007). Given this demographic disparity, age was systematically incorporated as a covariate in all subsequent multivariable GEE analyses to adjust for its potential confounding effects.

**TABLE 1:** Demographic and clinical baseline characteristics of the study population.

Variable	Healthy controls (*n* = 40)	FCAD GROUP (*n* = 40)	Statistic	*p*‐value
Age (years)	33.8 ± 6.4	39.1 ± 10.3	−2.772^a^	**0.007**
Gender			—	0.116^b^
Male	0 (0.0%)	4 (10.0%)		
Female	40 (100.0%)	36 (90.0%)		
BMI (kg/m^2^)	22.3 ± 3.3	23.0 ± 2.4	−1.026^a^	0.308

***Note***: Data are presented as mean ± standard deviation (SD) for continuous variables and number (percentage) for categorical variables. *p*‐values were calculated using two‐tailed tests.

**Abbreviations**: FCAD, Facial Corticosteroid Addiction Dermatitis; BMI, Body Mass Index; N/A, Not Applicable.

^a^ Calculated using independent samples *t*‐test.

^b^ Calculated using Fisher's exact test (due to cell counts < 5). Bold values indicate statistical significance (*p <* 0.05).

### 3.2 Site‐Specific Morphological and Functional Differences

Univariate analysis revealed that FCAD lesional areas exhibited significant differences from healthy skin concerning both ultrasonographic morphology and skin barrier function (Table 2).

**TABLE 2:** Univariate analysis of site‐specific ultrasonic and physiological factors associated with FCAD lesional sites compared to healthy sites.

Variable	Healthy sites (*n* = 160)	FCAD lesional sites (*n* = 119)	OR (95% CI)	*p*‐value
Ultrasonic features				
Entrance echo layer thickness (mm)	0.176 ± 0.013	0.182 ± 0.016	1.38 (1.05–1.81)	**0.021**
SLEB thickness (mm)	0.096 ± 0.012	0.106 ± 0.013	1.92 (1.39–2.66)	**< 0.001**
Blood Flow (PDI), *n* (%)				
Low/moderate flow (Grade 0–2)^a^	127 (79.4%)	18 (15.1%)	1.00 (Reference)	—
High flow (Grade 3)	33 (20.6%)	101 (84.9%)	21.59 (9.15–50.97)	**< 0.001**
Physiological metric				
TEWL (g/m^2^h)	22.1 ± 6.8	25.6 ± 7.4	1.07 (1.02–1.13)	**0.004**

*Note*: Data are presented as mean ± standard deviation (SD) for continuous variables and number (percentage) for categorical variables.

Abbreviations: CI, confidence interval; FCAD, facial corticosteroid addiction dermatitis; OR, odds ratio; PDI, power Doppler imaging; SLEB, subepidermal low‐echogenic band; TEWL, transepidermal water loss.

bold values indicate statistical significance (P < 0.05).

Statistical Analysis: Univariate analysis was performed using Generalized Estimating Equations (GEE) with a binomial distribution and logit link function to account for intra‐subject clustering of the 4 facial sites. An independent working correlation structure with robust standard errors (Huber‐White sandwich estimator) was employed for parameter estimation.

Variable Definitions: Odds Ratios (OR) for epidermis and SLEB thickness represent the risk increase per 0.01 mm increment in thickness. High blood flow is defined as PDI Grade 3 (marked hypervascularity), relative to low/moderate blood flow (Adler Grades 0–2).

^a^ Remark: Although the physiological PDI category is defined as Adler Grades 0–2, no Grade 0 signals were observed in the current study cohort.

#### 3.2.1 HFUS Morphology and Hemodynamics

HFUS (22 MHz) demonstrated that the SLEB thickness in lesional areas was significantly greater than that in the healthy control group (0.106 ± 0.013 mm vs. 0.096 ± 0.012 mm; *p <* 0.001). Site‐specific analysis further confirmed that this pathological thickening was consistently significant across the cheek (*p* < 0.0001), forehead (*p* < 0.01), and chin (*p* < 0.05) regions. Qualitatively, using 0.12 mm (the upper limit of normal) as the threshold for pathological thickening, the incidence of a pathological SLEB in FCAD lesions was significantly higher than in HCs (21.0% vs. 5.6%; *p <* 0.001; OR = 4.46). This indicates that although the absolute difference in mean thickness between the groups is modest, SLEB thickening remains a prominent pathological feature in a substantial subset of patients (Figure 2A), objectively reflecting inflammatory edema within the superficial dermis.

FIGURE 2Comparative HFUS features and quantitative physiological analysis of FCAD versus HCs.(A) Ultrasound & PDI Evaluation: Panels A and B (FCAD Patient): (A) 2D grayscale ultrasound (22 MHz) revealing a distinct SLEB (indicated by markers) with a measured thickness of 0.13 mm, signifying pronounced dermal edema. (B) PDI showing marked blood flow signals (Grade 3), indicating active inflammation and rebound vasodilation. Panels C and D (Healthy Control): (C) 2D image showing a normal, uniform hyperechoic dermal structure without significant SLEB. (D) PDI displaying minimal to moderate physiological background flow (Grade 1–2), characteristic of normal facial vascularity, without marked hypervascularity. Scale bar = 1 mm. (B) Quantitative Analysis: Quantitative comparison of SLEB thickness and TEWL between FCAD lesions and HCs across three anatomical sites. SLEB Thickness: FCAD patients exhibited significantly increased SLEB thickness compared to controls, particularly in the Cheek (*p* < 0.0001), Forehead (*p* < 0.01), and Chin (*p* < 0.05). TEWL: A significant elevation in TEWL was observed in the Cheek region of FCAD patients (*p* < 0.0001), reflecting localized barrier impairment. Notably, TEWL elevations in the forehead and chin regions were not statistically significant (ns), highlighting the regional heterogeneity of barrier dysfunction.Data are presented as Mean ± SD. Statistical significance was determined using the Mann–Whitney U test: ns, not significant; * *p* < 0.05; ** *p* < 0.01; **** *p* < 0.0001.

**Figure:**
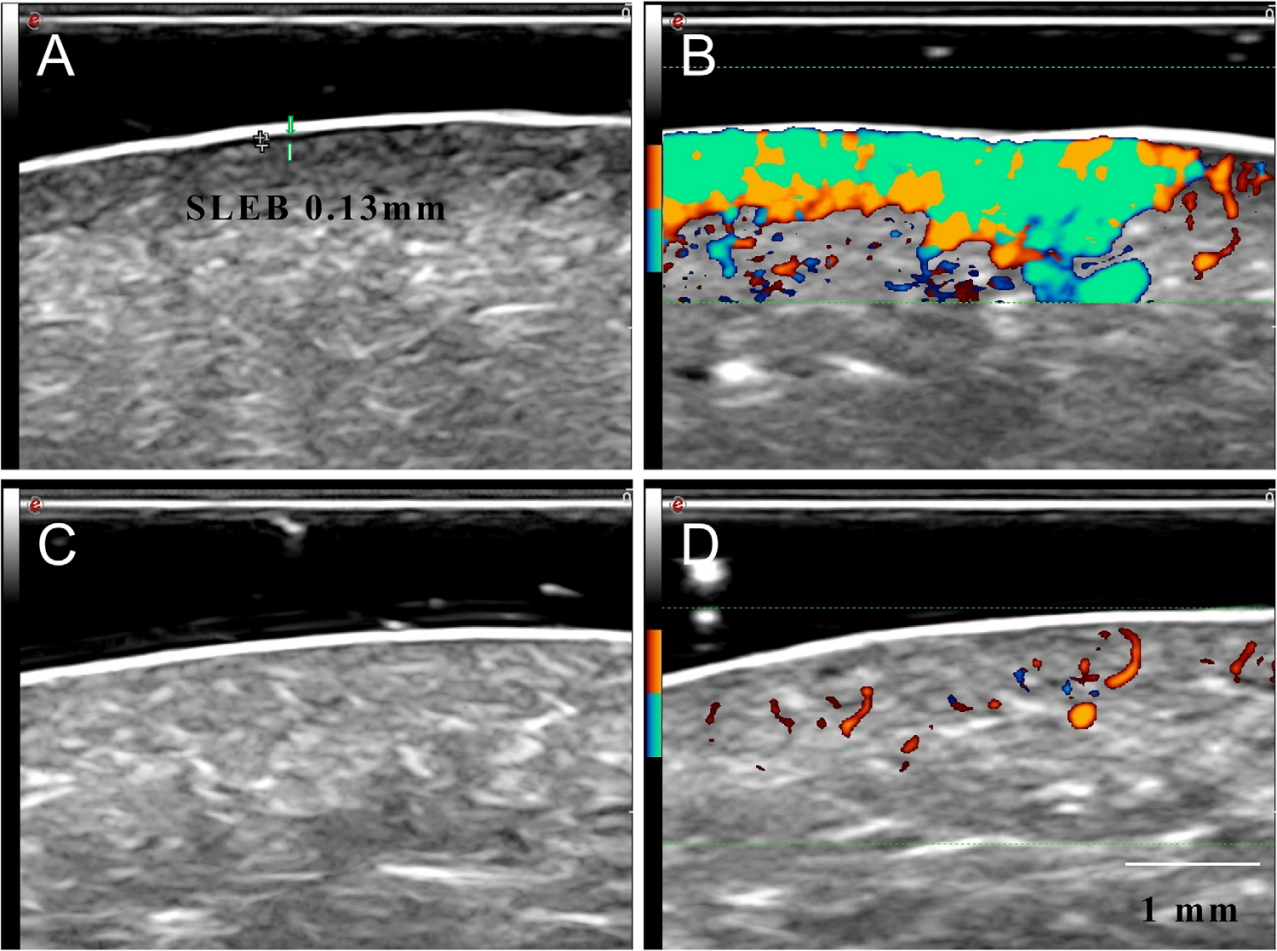

**Figure:**
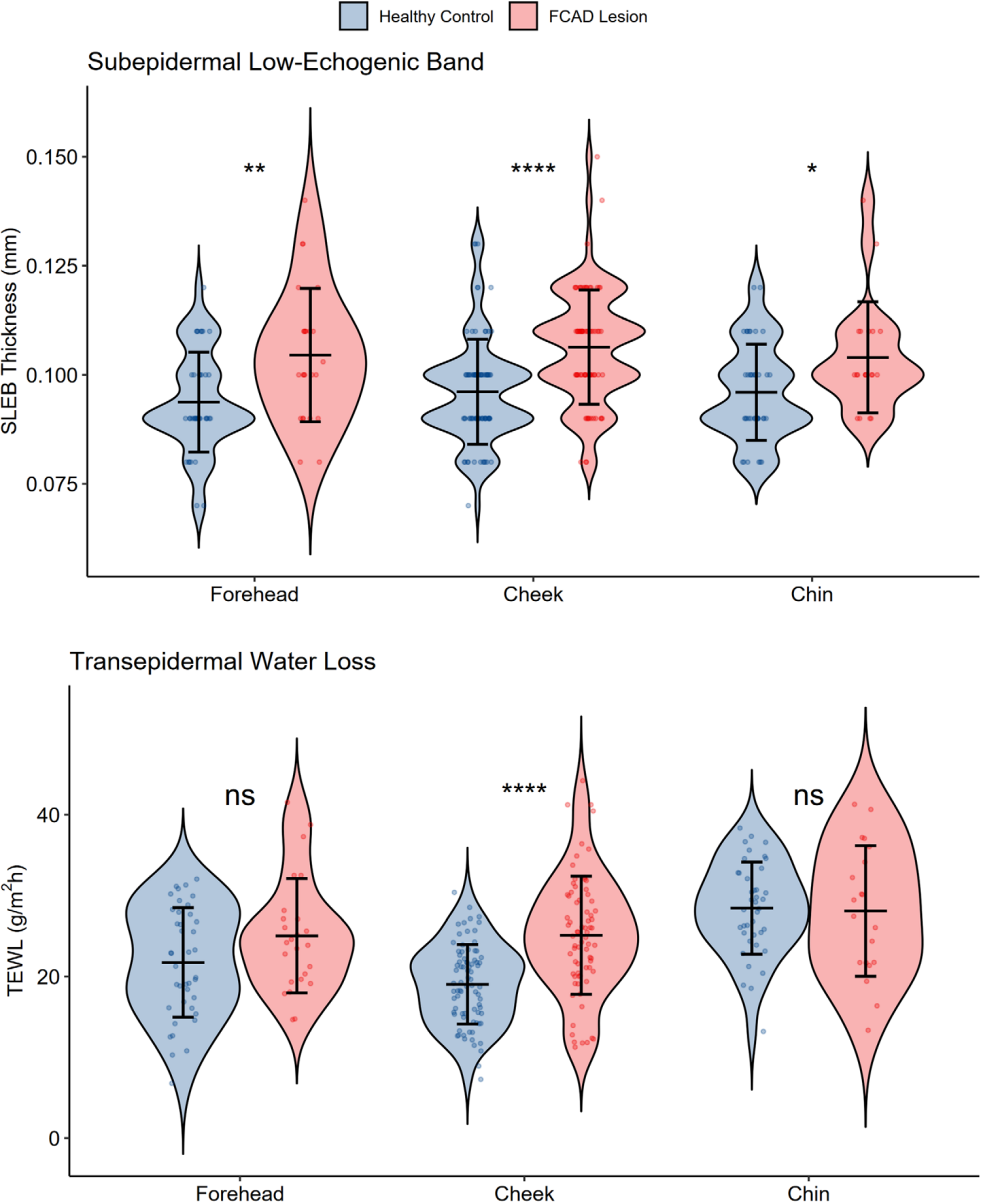

Regarding hemodynamics, PDI revealed a state of hyperperfusion within the FCAD lesional areas. Notably, no Adler Grade 0 (absent blood flow) signals were detected across any of the evaluated sites (including healthy controls). This finding is consistent with the inherently rich baseline vascularity characteristic of facial anatomy; consequently, the “physiological background flow” defined in our study (theoretically encompassing Adler Grades 0–2) was exclusively comprised of Adler Grade 1 and Grade 2 signals in the actual dataset. Furthermore, marked blood flow signals (Adler Grade 3) were detected in 84.9% (101/119) of the lesional sites, compared to only 20.6% (33/160) in the healthy control group, representing a highly significant difference (*p <* 0.001). The high frequency of these Grade 3 signals strongly suggests pronounced vasodilation and vascular proliferation within the papillary dermis.

#### 3.2.2 Barrier Function

TEWL values in the lesional areas were significantly elevated compared to the healthy control group (*p* = 0.004), indicating impaired skin barrier function. Notably, the TEWL data within the FCAD cohort displayed a considerably wider distribution range (Figure 2B), highlighting a profound degree of heterogeneity in the severity of barrier impairment across specific anatomical sites: while a highly significant elevation was observed in the cheek region (*p* < 0.0001), the differences in the forehead and chin regions were not statistically significant (ns).

#### 3.2.3 Sensitivity Analysis

To validate the robustness of the aforementioned findings, a sensitivity analysis utilizing PSM was conducted. After generating 28 pairs of matched cohorts with well‐balanced demographic profiles (Age: *p =* 1.000; BMI: *p =* 0.952), a site‐level comparative analysis was executed between 83 FCAD lesions and 112 healthy control sites. The results confirmed that significant intergroup differences persisted regarding entrance echo layer thickness, SLEB thickness, and Adler grade (all *p* < 0.01) even after matching (Supplementary Table S1). Notably, while the overall TEWL elevation trended higher, it lost statistical significance following rigorous GEE adjustment for intra‐subject clustering (*p* = 0.120). This specific morphological‐functional divergence further reinforces our core finding: FCAD is fundamentally a vascular‐driven dermatosis rather than a primary barrier‐defect disorder.

### 3.3 Independent Predictors for FCAD Lesion Identification

After adjusting for intra‐subject clustering effects and age using a generalized estimating equation (GEE) model, multivariable analysis identified four independent predictors significantly associated with the identification of the FCAD pathological state (Table 3).

**TABLE 3:** Multivariable analysis of independent anatomical, ultrasonic, and physiological predictors for the identification of FCAD lesions.

Variable	β (Coeff)	SE	aOR (95% CI)	*p*‐value
Site				
Forehead	Reference	—	1.00	—
Cheek	−0.372	0.439	0.69 (0.29–1.63)	0.396
Chin	−1.038	0.398	0.35 (0.16–0.77)	**0.009**
Ultrasound				
SLEB (per 0.01 mm increase)^b^	0.565	0.203	1.76 (1.18–2.62)	**0.005**
PDI (Grade 3 vs. Grade 0–2)^a^	2.969	0.554	19.46 (6.57–57.64)	**< 0.001**
Physiological				
TEWL (g/m^2^h)	0.105	0.035	1.11 (1.04–1.19)	**0.002**
Demographics				
Age (years)	0.075	0.034	1.08 (1.01–1.15)	**0.025**

**Abbreviations**: aOR, adjusted odds ratio; CI, confidence interval; PDI, Power Doppler Imaging; SE, robust standard error; SLEB, subepidermal low‐echogenic band; TEWL, transepidermal water loss.

Statistical Analysis: Multivariable analysis was performed using Generalized Estimating Equations (GEE) with an independent working correlation structure to account for intra‐subject clustering. Robust standard errors (SE) were used for parameter estimation.

Adjusted: The model was adjusted for age as a potential confounder.

^a^ High blood flow is defined as PDI Grade 3 (marked hypervascularity), relative to Low/Moderate blood flow (Adler Grades 0–2).

^b^ The Odds Ratio represents the risk increase per 0.01 mm increment in SLEB thickness.

#### 3.3.1 Vascularity

Marked blood flow signals (Adler Grade 3) demonstrated distinct group separation, emerging as the strongest independent correlate. Although the confidence interval was relatively wide due to the quasi‐complete separation of the data distribution, the effect size remained highly significant (adjusted odds ratio [aOR] = 19.46, 95% CI: 6.57–57.64; *p <* 0.001).

#### 3.3.2 Dermal Inflammation

SLEB thickness was positively correlated with the probability of identifying the FCAD pathological state. For every 0.01 mm increment in thickness, the odds of positive identification by the model increased by 76% (aOR = 1.76, 95% CI: 1.18–2.62; *p =* 0.005).

#### 3.3.3 Barrier Function

The TEWL value also served as a significant independent predictor (aOR = 1.11, 95% CI: 1.04–1.19; *p =* 0.002).

#### 3.3.4 Anatomical Site

The analysis revealed significant site‐specific heterogeneity in pathological features. Holding other morphological and functional parameters constant, the chin was associated with a significantly lower risk of being identified as positive compared to the forehead (the reference group) (aOR = 0.35, 95% CI: 0.16–0.77; *p =* 0.009), suggesting that this anatomical subunit possesses a distinct threshold for pathological deviation.

### 3.4 Construction and Validation of the Multimodal Lesion Identification Nomogram

Building upon the independent predictors identified above, this study constructed a multimodal quantitative assessment nomogram integrating anatomical site, SLEB, PDI blood flow, TEWL, and age (Figure 3). To utilize the nomogram, the patient's value for each variable is located on its corresponding axis, and a vertical line is drawn upward to the “Points” axis to determine the variable's score. The scores are summed to obtain the “Total Points,” and a vertical line is drawn downward to estimate the individual probability. Since the model was developed against HCs, this probability serves as a quantitative metric of pathological deviation (e.g., vascular rebound, structural edema) rather than a differential diagnostic probability against other dermatoses.

**FIGURE 3:**
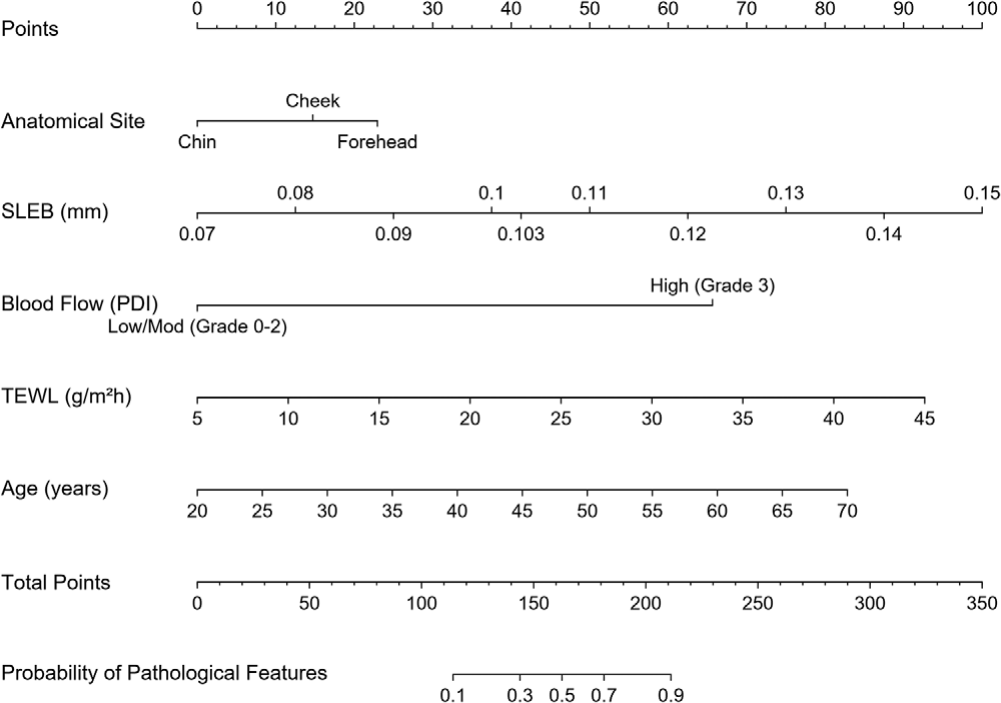
Multimodal quantitative assessment nomogram for FCAD integrating anatomical and ultrasonographic parameters. The nomogram was constructed based on the GEE model. *Note*: For “Blood Flow (PDI)”, the classification is dichotomized: ‘0’ represents physiological background flow (Adler Grades 0–2), and ‘1’ represents High Flow (Adler Grade 3, rebound vasodilation). Notably, while the reference range for Low/Moderate flow includes Adler Grade 0, no Grade 0 signals were observed in the current study cohort. Abbreviations: SLEB, Subepidermal Low‐Echogenic Band; TEWL, Transepidermal Water Loss; PDI, Power Doppler Imaging.

#### 3.4.1 Model Discrimination

ROC curve analysis revealed that the model possesses excellent discriminative performance, yielding an overall area under the curve (AUC) of 0.905 (95% confidence interval [CI]: 0.863–0.940). To optimize the model's clinical utility, the optimal predicted probability cutoff was identified as 0.441 by maximizing the Youden index. At this specific threshold, the model demonstrated robust efficacy in differentiating FCAD lesions from healthy skin, achieving a sensitivity of 85.7% and a specificity of 86.9%. Furthermore, based on the disease prevalence within the current study cohort, the PPV was 82.9%, and the NPV was 89.1%. These metrics indicate that the nomogram operates with notably low false‐positive and false‐negative rates.

#### 3.4.2 Subgroup Analysis and Robustness

Site‐specific subgroup analyses further substantiated the model's robustness: the forehead yielded the highest AUC (0.927), followed by the cheek (0.897) and the chin (0.879) (Figure 4). Pairwise comparisons utilizing the DeLong test revealed no statistically significant differences in AUC among the various anatomical subunits (forehead vs. chin, *p =* 0.471; forehead vs. cheek, *p =* 0.539; cheek vs. chin, *p =* 0.759). These findings confirm that, despite the inherent physiological heterogeneity of the face, the model maintains consistent and stable pathological identification efficacy across distinct anatomical regions.

**FIGURE 4:**
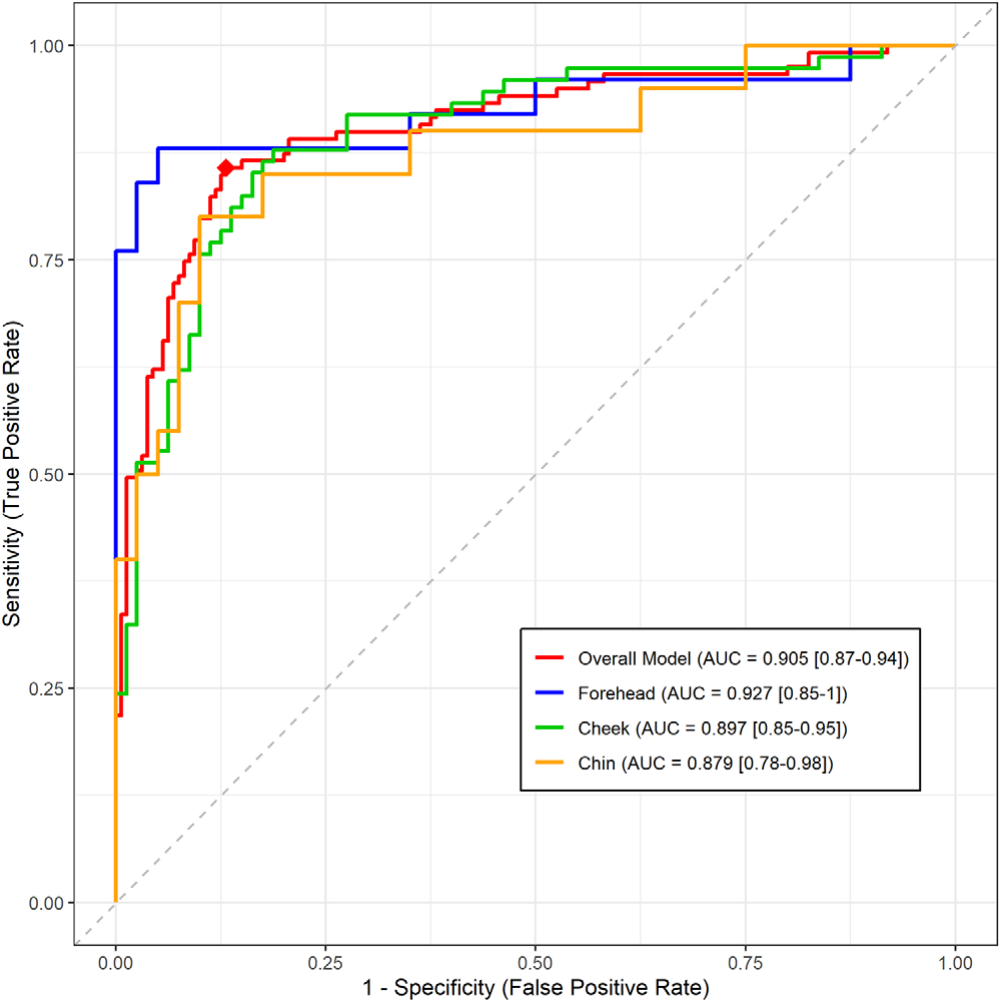
ROC curves illustrating the discriminative performance of the multimodal model. The curves depict the discriminatory ability across different anatomical datasets. The Overall Model (Red line) achieved an AUC of 0.905 (95% CI: 0.863–0.940). Subgroup analyses demonstrated robust performance for the Forehead (Blue), Cheek (Green), and Chin (Orange). The solid diamond marker on the overall curve represents the optimal pathological identification threshold (Youden index), corresponding to a sensitivity of 85.7% and specificity of 86.9%.

#### 3.4.3 Model Comparison and Incremental Value

To verify whether the multimodal integration offers superior discriminatory capacity for pathological identification compared to a single‐vascular‐parameter model, their respective ROC curves were compared. The single‐parameter model, comprising exclusively PDI (the dichotomized blood flow signal), yielded an AUC of 0.821. In contrast, the integrated nomogram achieved a significantly higher AUC (0.905; *p <* 0.05). This substantial enhancement (ΔAUC > 0.08) demonstrates that the incorporation of structural (SLEB) and functional (TEWL) parameters provides critical information regarding pathological features that a solitary hemodynamic indicator fails to capture.

#### 3.4.4 Calibration

The calibration curve (Figure 5) demonstrated high concordance between the predicted probabilities and the actual observed probabilities. The mean absolute error (MAE) was 0.021, indicating that the model is devoid of any significant overestimation or underestimation bias.

**FIGURE 5:**
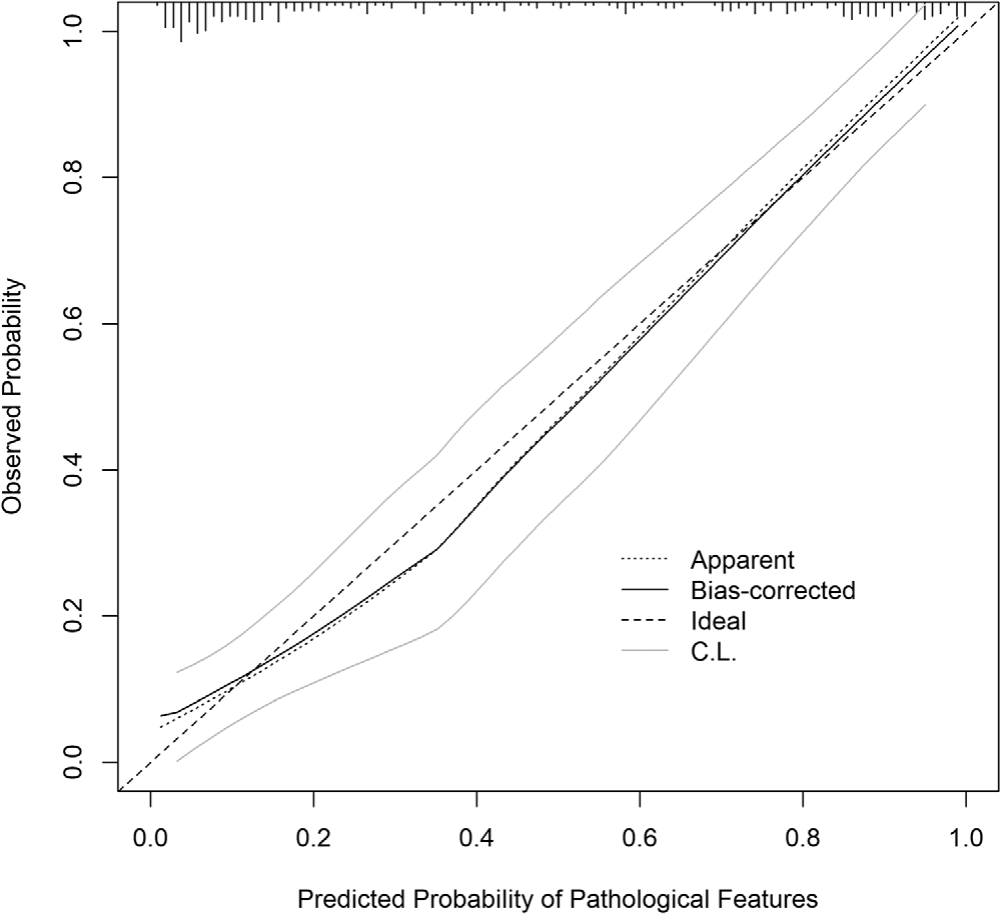
Calibration curve of the multimodal nomogram. The x‐axis represents the predicted probability of pathological features, and the y‐axis represents the actual observed probability. The diagonal dashed line represents the ideal prediction of a perfect model. The solid black line indicates the bias‐corrected performance, while the dotted line represents the apparent performance. Internal validation was performed using cluster‐based bootstrap resampling (*B* = 1000) to account for the intra‐subject clustering effect of multisite data. The MAE of 0.021 demonstrates high concordance between the predicted probabilities and actual observed outcomes, indicating no significant overestimation or underestimation bias.

#### 3.4.5 Clinical Benefit

DCA (Figure 6) revealed that across a broad range of threshold probabilities (10% to 90%), utilizing this multimodal nomogram for lesion identification consistently yielding a higher net benefit compared to both the “treat‐all” and “treat‐none” strategies, thereby highlighting its potential for clinical application.

**FIGURE 6:**
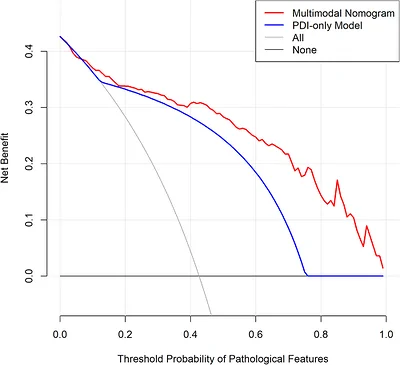
DCA of the multimodal nomogram. The y‐axis measures the net benefit. The red line represents the multimodal nomogram, while the blue line represents the single‐parameter PDI‐only model. The gray line represents the “Treat All” strategy (assuming all sites are pathological), and the horizontal black line represents the “Treat None” strategy. The decision curve demonstrates that the multimodal nomogram provides a higher net clinical benefit compared to both the PDI‐only model and the default strategies across a broad range of threshold probabilities (approximately 0.1 to 0.9).

## 4 Discussion

Past evaluations of FCAD have predominantly relied on the subjective clinical experience of dermatologists, often presenting diagnostic challenges due to phenotypic overlaps with rosacea and acne [2, 5]. However, resolving this complex differential diagnostic dilemma first requires establishing an objective, quantitative baseline for FCAD that is distinct from the physiological homeostasis of healthy skin [6]. Addressing the critical lack of objective assessment tools, this study developed and validated the first multimodal nomogram integrating anatomical heterogeneity [10], 22‐MHz HFUS, PDI, and TEWL. In differentiating FCAD lesions from healthy skin, our model demonstrated excellent discriminative performance (AUC = 0.905), significantly outperforming traditional single‐modality assessment paradigms. Furthermore, DCA demonstrated that the model yields a high net clinical benefit across a broad range of threshold probabilities [20]. Consequently, this study provides a novel approach in the evaluation of FCAD, transitioning from experience‐based visual assessment towards a more objective, quantitative imaging framework.

The robust quantitative efficacy of this nomogram is driven by a multimodal synergy that successfully bridges the vascular, structural, and functional dimensions of the skin [7, 14, 16]. By accurately capturing the complex pathophysiological features of FCAD, this integrated approach effectively overcomes the assessment blind spots inherent to single‐modality evaluations.

Primarily, anomalies within the vascular dimension represent the most prominent feature captured by the model. High‐grade PDI signals (Adler Grade 3) emerged as the strongest independent predictor (aOR = 19.46), functioning as a specific vascular alarm for the rebound phenomenon following corticosteroid withdrawal [21, 22]. From a pathophysiological perspective, this finding strongly supports the “nitric oxide (NO) rebound hypothesis”: prolonged topical application of glucocorticoids induces vascular endothelial dysfunction, suppressing the synthesis of endothelial nitric oxide (NO) [3], thereby locking local microvessels in a sustained state of vasoconstriction. Upon cessation of the drug, this inhibition is abruptly lifted; the accumulated NO precursors, coupled with a compensatorily upregulated receptor system, trigger an explosive rebound vasodilation [3, 23]. This unique hemodynamic pattern also serves as a critical differentiator from allergic contact dermatitis (ACD), which is predominantly characterized by immune‐mediated inflammatory infiltration and rarely exhibits such intense and diffuse vascular rebound signals.

Furthermore, the structural and functional dimensions provide the model with critical supplementary information. SLEB precisely quantifies the structural disruption induced by dermal edema [7, 24], whereas TEWL sensitively detects macroscopically imperceptible impairments in epidermal barrier function [14]. To mitigate the risk of the model over‐relying on a single vascular signal, a sensitivity analysis was conducted by removing the PDI variable. The results demonstrated that even with the complete exclusion of hemodynamic parameters, a simplified model based solely on anatomical site, SLEB, and TEWL retained robust discriminative efficacy (AUC = 0.806). This consistent performance confirms that the pathological alterations of FCAD extend beyond mere vasodilation; its hallmark dermal structural damage (SLEB) and barrier function disruption (TEWL) possess independent pathological indicator values [3, 25], playing a pivotal role, particularly in non‐acute phases or in cases exhibiting subtle vascular responses.

A major innovation of this model lies in the incorporation of the anatomical site as an independent correction factor. This design successfully mitigates the confounding effects of the inherent physiological heterogeneity of facial skin on objective assessments [10, 26, 27]. Consequently, the nomogram equips clinicians with a site‐adjusted, robust, and objective adjunctive decision‐making support system. This approach obviates the need to establish fragmented diagnostic criteria for disparate anatomical regions, thereby minimizing subjective bias in clinical evaluations to the greatest extent possible.

**At the microstructural level**: Thickening of the SLEB (aOR = 1.76) emerged as a critical hallmark of dermal structural damage. While SLEB thickening is traditionally regarded as a classic feature of intrinsic and photo‐induced cutaneous aging (particularly in sun‐exposed facial areas of older individuals) [28], within the pathophysiological context of FCAD, it predominantly reflects inflammatory edema localized in the papillary dermis [7, 24]. It is crucial to acknowledge that while our PSM successfully adjusted for statistical age imbalances between the cohorts, it does not resolve the inherent lack of specificity of SLEB as an isolated biomarker. Because SLEB can occur independently of FCAD as a physiological aging phenomenon, its association with FCAD must be interpreted with caution. However, this biological overlap perfectly underscores the necessity of our multimodal approach: while an older healthy individual may exhibit a baseline SLEB due to photoaging, they typically lack the concurrent marked vascular rebound (PDI Grade 3) and barrier disruption (TEWL) that characterize the active FCAD state. Importantly, although the absolute difference in mean SLEB thickness between the cohorts was numerically modest, the robust statistical significance of this variance underscores a pervasive trend of pathological alterations within the dermal matrix among patients with FCAD. Consequently, this finding carries significant clinical implications: it confirms that underlying, macroscopically imperceptible dermal structural damage may already be present, even in lesions exhibiting only mild macroscopic erythema.

### 4.1 Functional and Anatomical Dimensions

Serving as a critical adjunct to the aforementioned imaging biomarkers, TEWL functioned as an independent indicator of physiological function (aOR = 1.07) [14]. Our data demonstrate that the disruption of barrier function constitutes a distinct pathophysiological dimension, and its severity does not necessarily parallel the intensity of vasodilation (PDI) in a linear fashion. This phenomenon of morphological‐functional dissociation suggests that corticosteroid‐induced damage to the stratum corneum often exhibits a delayed recovery; specifically, occult barrier dysfunction may persist long after the apparent resolution of the vascular response (i.e., erythema) [29, 30]. By integrating these three complementary biological signals, the multimodal model establishes a comprehensive, multidimensional assessment framework, thereby effectively mitigating the risk of false negatives associated with relying solely on morphological features or clinical symptomatology [31].

Notably, the generalized estimating equation (GEE) model quantified a significant anatomical site effect: whereas the cheek and forehead regions exhibited comparable pathological risks, the chin area exhibited a phenomenon of relative sparing (aOR = 0.35 compared to the forehead). This statistical outcome is highly consistent with the sample distribution profile of this cohort. The incidence of lesions in the chin region was the lowest (accounting for only 16% of total lesions); this phenomenon (i.e., relative perioral sparing) inherently resulted in a relatively small effective sample size for this specific anatomical subunit [21]. Nevertheless, even with this constrained sample size, multivariable analysis revealed a robust and highly significant statistical effect for this region (aOR = 0.35; *p =* 0.009). This remarkably low odds ratio strongly suggests that the chin region possesses a higher pathological threshold. Consequently, this anatomical subunit is typically implicated only in patients with more severe, generalized disease presentations, a finding that further supports its unique anatomical resilience [26].

### 4.2 Hierarchical Revelation of Pathophysiological Mechanisms

Regarding the pathophysiological contributions of the multimodal parameters, this study observed that the adjusted odds ratio (aOR) for the PDI signal reached a notable 19.46. This elucidates the inherent hierarchy of pathological identification within the multimodal nomogram: vasomotor dysfunction (PDI) >> dermal structural edema (SLEB) > epidermal barrier impairment (TEWL). This pronounced disparity in predictive weights (aOR ∼19 vs. ∼1.1) strongly reflects the core pathological attribute of FCAD: it is a vascular‐driven condition characterized predominantly by NO‐mediated rebound vasodilation [22], rather than a primary barrier‐defect disorder. This hallmark presents a distinct pathophysiological contrast to atopic dermatitis (AD). Previous studies have established that the pathogenesis of AD is primarily driven by epidermal barrier impairment, with vascular responses typically occurring as secondary phenomena [14, 32].

Consequently, the current model successfully quantifies and establishes the unique vascular‐dominant pathophysiological profile of FCAD. The robust evidence of rebound vasodilation—manifested as high‐grade PDI signals evaluated via the Adler grading system—not only highlights its fundamental mechanistic divergence from AD but also suggests its potential utility as a critical differential biomarker. In future investigations, this distinct hemodynamic signature could facilitate the precise differentiation of FCAD from other facial dermatoses primarily characterized by barrier impairment [22, 32].

### 4.3 Limitations and the Necessity of a Multimodal Approach

Nevertheless, PDI signals warrant cautious interpretation. A notable finding of this study was the complete absence of Adler Grade 0 (absent blood flow) signals across all subjects, including the HCs. This zero incidence provides critical anatomical evidence confirming the inherently high baseline perfusion of facial skin [33]. Empirically, this substantiates our physiological calibration of the Adler grading system: in facial ultrasonographic assessments, the physiological baseline must be adjusted upward from the traditional Grade 0 to encompass Grades 1 and 2. Notably, our data revealed that approximately 20.6% of the healthy control sites also exhibited marked blood flow signals (Adler Grade 3). Although Grade 2 was established as the upper limit of the normal physiological baseline, the emergence of Grade 3 signals in a subset of HCs demonstrates the high sensitivity of HFUS in detecting microvascular flow—along with its inherent limitations. Specifically, the 22‐MHz transducer's capability to detect low‐velocity microcirculation within the dermis may capture transient physiological vasodilation that can be misinterpreted as pathological hyperperfusion. During examinations, emotional stress or the mechanical and thermal stimuli elicited by transducer contact can readily provoke such transient responses in the face.

During examinations, emotional stress or the mechanical and thermal stimuli elicited by transducer contact can readily provoke transient physiological vasodilation in the face. These transient responses are subsequently captured by PDI and can be misinterpreted as marked blood flow [34]. On a solitary PDI scan, differentiating this from the inflammation‐mediated, persistent pathological vascular proliferation observed in patients with FCAD is challenging. Ultimately, this finding underscores the necessity of constructing a multimodal nomogram [31]. While relying exclusively on PDI is prone to yielding false positives, within the multimodal framework, these false‐positive healthy sites—lacking concurrent SLEB thickening and TEWL elevation—were accurately adjusted and correctly classified as healthy by the model. This demonstrates the nomogram's specificity in distinguishing between transient functional hyperemia and persistent organic pathological alterations.

### 4.4 Clinical Translational Value

Beyond the mechanistic insights discussed above, a core translational value of this study lies in the development of a highly practical, visual decision‐support tool for clinical settings. The complex generalized estimating equation (GEE) regression model was successfully translated into an intuitive nomogram (Figure 3). This allows dermatologists to simply sum the designated scores for the anatomical site, SLEB, PDI, and TEWL to rapidly compute the probability of FCAD. This graphical representation converts complex statistical coefficients into actionable, individualized clinical probabilities, thereby significantly lowering the barrier to its clinical application [19, 35]. Furthermore, the model achieves an optimal balance in discrimination: a sensitivity of 85.7% ensures a low false‐negative rate for occult lesions, whereas a specificity of 86.9% effectively mitigates the risk of misinterpreting physiological vascular fluctuations as pathological alterations.

DCA further demonstrated that across a broad continuum of threshold probabilities, the multimodal nomogram consistently yielded a significantly higher net clinical benefit compared to either the treat‐all or treat‐none strategies (Figure 6) [20, 36]. These findings demonstrate the dual clinical utility of this tool in striking a delicate balance between minimizing missed diagnoses and preventing overtreatment.

### 4.5 Methodological Advantages and Statistical Adjustments

Methodologically, this study addressed a prevalent statistical pitfall in contemporary multisite dermatological research. Previous literature has frequently treated measurement data derived from distinct facial regions (e.g., the forehead and cheeks) of the same patient as independent observations, relying on traditional analysis of variance (ANOVA) or Student's *t*‐tests for analysis. This practice violates the assumption of independence in statistics; by neglecting intra‐subject correlations, it inevitably leads to an artificial inflation of Type I errors (i.e., false‐positive rates) [37].

By employing GEE, the clustered structure of the data (i.e., the nested nature of four anatomical sites per patient) was appropriately accounted for. This modeling approach ensures that the derived site‐specific effect sizes, such as the lower risk associated with the chin, reflect biological divergences rather than statistical artifacts arising from intra‐subject variability [18, 37]. Consequently, this analytical strategy serves as a methodological reference for future dermatological investigations involving anatomical site specificity, advocating for the adoption of a robust statistical framework that accounts for the physiological interconnectedness of facial subunits [10].

### 4.6 Clinical Implementation Strategy

To facilitate the practical application of the model at the patient level, this study proposes a standardized worst‐lesion‐first strategy. Given the heterogeneous distribution of FCAD lesions across various facial subunits, it is recommended that clinicians select the most clinically severe site (i.e., the area exhibiting the most pronounced erythema or the most intense symptoms) as the index lesion for measurement [21, 38]. If the nomogram score at this specific site indicates a high risk, it confirms the presence of active FCAD pathological alterations. Furthermore, the utility of this quantitative tool extends beyond a single diagnostic assessment; its greater value lies in longitudinal monitoring. By tracking the score trajectory of the same index lesion throughout the treatment course, dermatologists can objectively evaluate the resolution kinetics of the corticosteroid withdrawal response, thereby providing a robust quantitative rationale for the precise de‐escalation of therapeutic regimens [25, 31].

The limitations of the present study must be acknowledged.

**Single‐center design and sample size**: First, given the single‐center design, the sample size is relatively limited. This constraint introduces statistical fluctuations in the parameter estimations of certain strong predictors. For instance, although the PDI signal yielded a high effect size (aOR = 19.46), it is accompanied by a wide confidence interval. This statistical phenomenon is primarily attributable to the quasi‐complete separation of the data distribution [39, 40]; specifically, high‐grade blood flow signals are densely concentrated within clinically overt FCAD lesions yet remain relatively infrequent among HCs. While this underscores the robust discriminative capacity of the indicator as a pathological feature, it also suggests that future validation studies must expand their sample sizes to secure more precise parameter estimates. Furthermore, the characteristic relative perioral sparing observed in FCAD resulted in a significantly smaller volume of positive samples in the chin region compared to the cheeks and the forehead. Although the GEE model effectively adjusted for this imbalanced data structure, it may still contribute to wider confidence intervals for predictive parameters corresponding to this specific anatomical subunit. Nevertheless, to validate the overall robustness of the findings, a sensitivity analysis utilizing PSM was conducted (Supplementary Table S1). The results confirmed that, even after rigorously controlling for demographic confounders, significant pathological divergences persisted within the FCAD cohort [17]. This confirms that the primary conclusions of the research are not compromised by the potential fluctuations inherent to a single‐center sample.

**External validity and prevalence dependence**: Second, the external validity of the proposed model warrants further verification. Although rigorous internal validation was executed via cluster‐based bootstrap resampling (*B* = 1000), adherence to the Transparent Reporting of a multivariable prediction model for Individual Prognosis or Diagnosis (TRIPOD) statement dictates that the generalizability of this nomogram must be externally validated across larger, multicenter, and multiethnic cohorts [41]. Additionally, the PPV reported herein is intrinsically dependent on the specific prevalence of FCAD within the study cohort (42.6%). In primary care settings characterized by lower disease prevalence, the PPV might correspondingly decrease. However, the high sensitivity (85.7%) and specificity (86.9%) demonstrated by the model indicate that its discriminative performance remains stable and fundamentally independent of disease prevalence [42].

**Control group design**: Third, the control group design intentionally selected healthy volunteers to serve as the reference baseline, explicitly excluding confounding dermatoses such as rosacea. This approach aimed to establish a definitive reference baseline for FCAD relative to physiological homeostasis. Before addressing complex differential diagnostic challenges, it is essential to accurately quantify the precise degree of pathological deviation inherent to FCAD itself. Consequently, the high specificity reported by the model should be interpreted as the tool's efficacy in identifying a pathological (non‐healthy) state [41]. The quantitative hallmarks established herein (e.g., marked PDI rebound, SLEB thickening) will provide the essential parametric foundation for subsequent differential diagnostic investigations incorporating disease control groups [43].

**Measurement bias and protocol**: Fourth, the present study is subject to potential measurement bias. Because the quintessential clinical signs of FCAD (e.g., diffuse erythema) are macroscopically visible, complete masking of the subjects' cutaneous status was unattainable for the sonographer during the examination. This visibility may have introduced a degree of observer expectation bias during the measurement of SLEB thickness. To mitigate this effect, a standardized anatomical positioning protocol was strictly enforced. Specifically, irrespective of lesion severity, data acquisition was systematically performed at predetermined coordinates within each anatomical subunit (e.g., the geometric center of the cheek, the midline of the glabella), rather than through subjective sonographic hunting for the region exhibiting the strongest signal. This systematic sampling strategy ensures that the analyzed lesional sites genuinely reflect the average pathological state of that anatomical region, rather than artificially selected extreme values, thereby safeguarding the objectivity of the model [10]. Additionally, transient hemodynamic fluctuations induced by examination anxiety during the procedure are difficult to eliminate entirely, which may account for the subset of high PDI signals observed within the healthy control group. This phenomenon indirectly highlights the limitations of relying exclusively on a solitary hemodynamic indicator in complex clinical scenarios [44]. Furthermore, due to the single‐center, single‐operator design—while maximizing the internal consistency of the measurement criteria—no formal inter‐rater reliability assessment was conducted. Additionally, the use of a 22‐MHz ultrasound transducer presents an inherent methodological trade‐off. While this frequency facilitates the capture of hemodynamics via PDI, it lacks the superior spatial resolution provided by ultrahigh‐frequency transducers (>50 MHz), which are better suited for the precise anatomical segmentation of epidermal and superficial dermal layers. Consequently, the resolution limits of our imaging system may introduce minor measurement uncertainties regarding the entrance echo layer and SLEB thickness. Future studies should ideally employ ultrahigh‐frequency probes to further validate and refine these quantitative benchmarks. Future research should prioritize multicenter validation incorporating inter‐rater reliability evaluations and investigate the integration of automated image processing and deep‐learning‐based segmentation models to minimize operator‐dependency and further enhance the reliability and reproducibility of the analysis [45].

**SLEB measurement precision**: Fifth, the measurement precision of the SLEB warrants cautious interpretation regarding the marginal intergroup numerical variance (∼0.01 mm). While the absolute mean difference is numerically small, the highly significant *p*‐value (< 0.001) obtained via GEE, which accounts for intra‐subject clustering, indicates that this shift represents a systematic pathological deviation rather than random measurement noise [46]. To mitigate the impact of inherent 22‐MHz resolution limits, we specifically implemented a dichotomization strategy (defining SLEB ≥ 0.12 mm as pathologically thickened). This approach shifts the focus from micron‐level thickness discrepancies to the presence of clinically relevant dermal edema, effectively neutralizing the influence of minor boundary delineation uncertainties. As demonstrated in our sensitivity analysis, even after applying this stringent threshold, the pathologically thickened SLEB remained a robust diagnostic indicator (OR = 4.46), confirming that the observed findings are not only statistically significant but also clinically meaningful in characterizing the FCAD pathological state. These data confirm that superficial dermal edema is an inherent pathological hallmark of FCAD, suggesting that its clinical application should emphasize its utility as a qualitative imaging biomarker rather than relying exclusively on absolute measurements [7, 47]. Furthermore, a limitation of the current study design is the absence of disease‐control groups. While our current study focused on establishing the baseline imaging characteristics of FCAD versus healthy skin, the clinical overlap with other facial dermatoses such as rosacea and acne remains a critical challenge. Consequently, ongoing work is actively recruiting a broader range of patients with morphologically similar dermatoses to refine the nomogram and enhance its ability to navigate complex differential diagnoses, thereby solidifying its real‐world clinical utility.

**Analytical scope and future directions**: Finally, the analytical scope of the present study was strictly confined to clinically overt lesional areas. It is acknowledged that the pathophysiological continuum of FCAD extends beyond overt lesions, encompassing subclinical barrier impairment in macroscopically normal‐appearing skin—the insidious root cause of recurrent exacerbations [48]. While the multimodal quantitative model established herein successfully addresses the clinical imperative for the objective assessment of active pathological states, future investigations must integrate advanced modalities, such as noninvasive skin lipidomics, to decode the microenvironmental foundations driving anatomical resilience and to extend the evaluation to lesions in the quiescent or latent phases [49].

## 5 Conclusion

This study addresses the critical clinical gap of objective assessment for FCAD by validating a multimodal approach that integrates anatomical site specificity. A nomogram incorporating HFUS, skin barrier function (TEWL), and anatomical site weights was successfully established, demonstrating excellent discrimination and substantial net clinical benefit. Core findings underscore the pronounced relative perioral sparing of the chin region, establish high‐grade PDI signals as a primary indicator of the post‐withdrawal rebound phenomenon, and highlight the synergistic value of SLEB thickness and TEWL across structural and functional dimensions.

Theoretically, this research challenges the traditional homogenized facial assessment paradigm by proposing a site‐adjusted quantitative framework. In clinical practice, this visual decision‐support tool is intended to serve as an adjunctive mechanism for quantitative assessment and disease monitoring, mitigating subjective empirical biases. While limited by its single‐center design, future research should focus on multicenter validation and exploring subclinical monitoring potentials. Overall, this site‐specific, multimodal system represents a valuable step toward more objective dermatologic imaging in FCAD management.

## Funding

This work was supported by the Key Medical Discipline Construction Project of Jiading District Health System, Shanghai (Dermatology) (Grant No. XK202406); and the Key Discipline of Integrated Traditional Chinese and Western Medicine (Dermatology) under the Public Hospital Reform and High‐Quality Development Demonstration Project of Jiading District, Shanghai (Grant No. JDZXYJHZDXK04).

## Ethics Statement

The study was conducted in accordance with the Declaration of Helsinki. The research protocol was reviewed and approved by the Institutional Review Board of Ruijin Hospital Nanxiang Branch (Approval No.:2020006). All participants provided voluntary written informed consent prior to the commencement of the study.

## Conflicts of Interest

The authors declare no conflicts of interest.

## Supporting information




**Supporting File**: srt70388‐sup‐0001‐SuppMat.docx.

## Data Availability

The data that support the findings of this study are available from the corresponding author upon reasonable request.
