# Supplementary material for: Site‐Specific Characterization and Quantification of Facial Corticosteroid Addiction Dermatitis: A Multimodal Nomogram Integrating High‐Frequency Ultrasonography and Transepidermal Water Loss
Source: Skin Res Technol. 2026 Oct 12;32(10):e70388. doi: 10.1111/srt.70388 (PMC13656343; doi:10.1111/srt.70388)
Supplement: Supplementary file 1 [file SRT-32-e70388-s001.docx]

**Supplementary** **Table S1.** Comparison of demographic and clinical characteristics after Propensity Score Matching (PSM)

| **Variable** | **FCAD Group (n = 28)** | **Control Group (n = 28)** | **P-value** |
| --- | --- | --- | --- |
| Demographics |  |  |  |
| Age (years) | 34.0 ± 6.7 | 34.0 ± 6.5 | 1.000 |
| BMI (kg/m²) | 22.6 ± 1.9 | 22.7 ± 3.5 | 0.952 |
| Clinical Features | (n = 83 lesions) | (n = 112 sites) |  |
| Entrance echo layer thickness (mm) | 0.185 ± 0.015 | 0.175 ± 0.013 | **< 0.001** |
| SLEB thickness (mm) | 0.105 ± 0.012 | 0.097 ± 0.012 | **< 0.004** |
| Blood Flow (PDI), n (%) |  |  | **< 0.001** |
| Low/Moderate Flow (Grade 0–2) † | 16 (19.3%) | 97 (86.6%) |  |
| High Flow (Grade 3) | 67 (80.7%) | 15 (13.4%) |  |
| TEWL (g/m²h) | 24.7 ± 6.9 | 22.4 ± 6.8 | **0.120** |

**Note:** Data are presented as mean ± standard deviation (SD) or number (percentage).

Abbreviations: BMI, body mass index; FCAD, facial corticosteroid addiction dermatitis; PDI, power Doppler imaging; SLEB, subepidermal low-echogenic band; TEWL, transepidermal water loss.

**Statistical Analysis:** P-values were calculated using the independent samples t-test for continuous variables (age, BMI, entrance echo layer thickness, SLEB thickness, TEWL) and the chi-square test for categorical variables (PDI grade).

**Variable Definitions:** P-values for demographic variables (Age, BMI) were calculated using the independent samples t-test at the patient level (n = 28 vs. 28). For clinical features (entrance echo layer thickness, SLEB thickness, TEWL, and PDI grade) evaluated at the site level (n = 83 lesions vs. 112 healthy sites), P-values were calculated using Generalized Estimating Equations (GEE) to rigorously account for the intra-subject clustering effect of multi-site data.
